# The Effects of Transcranial Direct Current Stimulation in Patients with Mild Cognitive Impairment

**DOI:** 10.3390/neurolint15040092

**Published:** 2023-11-29

**Authors:** Matei Palimariciuc, Dan Cătălin Oprea, Ana Caterina Cristofor, Tudor Florea, Romeo Petru Dobrin, Irina Dobrin, Bogdan Gireadă, Radu Gavril, Iasmin Mawas, Andreea Cristina Bejenariu, Anton Knieling, Alin Ciobica, Roxana Chiriță

**Affiliations:** 1 Department of Medicine III, Faculty of Medicine, Grigore T. Popa University of Medicine and Pharmacy of Iasi, 700115 Iasi, Romania; matei.palimariciuc@umfiasi.ro (M.P.); oprea.dan-catalin@d.umfiasi.ro (D.C.O.); caterina.cristofor@umfiasi.ro (A.C.C.); florea.tudor@umfiasi.ro (T.F.); irina.dobrin@umfiasi.ro (I.D.); gireada_bogdan@d.umfiasi.ro (B.G.); radu_v_gavril@d.umfiasi.ro (R.G.); mawas.iasmin@gmail.com (I.M.); andreea_costana@yahoo.com (A.C.B.); roxana.chirita@umfiasi.ro (R.C.); 2 Institute of Psychiatry “Socola”, 36 Bucium Street, 700282 Iasi, Romania; 3 Institute of Forensic Medicine, 700455 Iași, Romania; anton.knieling@umfiasi.ro; 4 Forensic Science Department, Faculty of Medicine, Grigore T. Popa University of Medicine and Pharmacy of Iasi, 700115 Iasi, Romania; 5 Department of Biology, Faculty of Biology, Alexandru Ioan Cuza University, B-dul Carol I No. 11, 700506 Iasi, Romania; alin.ciobica@uaic.ro; 6 Academy of Romanian Scientists, Splaiul Independentei Nr. 54, Sector 5, 050094 Bucuresti, Romania; 7 Centre of Biomedical Research, Romanian Academy, B-dul Carol I No. 8, 700506 Iasi, Romania; 8 Preclinical Department, Apollonia University, Păcurari Street 11, 700511 Iași, Romania

**Keywords:** tDCS, mild cognitive impairment, neuromodulation, high-definition transcranial direct current stimulation

## Abstract

Transcranial direct current stimulation (tDCS) came into consideration in recent years as a promising, non-invasive form of neuromodulation for individuals suffering from mild cognitive impairment (MCI). MCI represents a transitional stage between normal cognitive aging and more severe cognitive decline, which appears in neurodegenerative diseases, such as Alzheimer’s disease. Numerous studies have shown that tDCS can have several useful effects in patients with MCI. It is believed to enhance cognitive functions, including memory and attention, potentially slowing down the progression of neurodegeneration and cognitive decline. tDCS is believed to work by modulating neuronal activity and promoting synaptic plasticity in the brain regions associated with cognition. Moreover, tDCS is generally considered safe and well-tolerated, making it an attractive option for long-term therapeutic use in MCI. However, further research is needed to determine the optimal stimulation parameters and long-term effects of tDCS in this population, as well as its potential to serve as a complementary therapy alongside other interventions for MCI. In this review, we included 16 randomized clinical trials containing patients with MCI who were treated with tDCS. We aim to provide important evidence for the cognitive enhancement using tDCS in patients with MCI, summarizing the effects and conclusions found in several clinical trials, and discuss its main mechanisms.

## 1. Introduction

### 1.1. Mild Cognitive Impairment

Mild cognitive impairment (MCI) was characterized in 1997 by Petersen et al. as being an abnormal function of the memory, more than in normal ageing, that does not impair general cognitive function and daily living activities, the patient being self-aware and complaining about the deficit in the absence of dementia [1].

In the fifth edition of the Diagnostic and Statistical Manual of Mental Disorders (DSM-V), not previously defined in earlier editions, MCI is referred to as a “Mild Neurocognitive Disorder” with the specification that there should be cognitive decline from a previous level of function in one or more of the six cognitive domains (complex attention, executive function, learning and memory, language, perceptual motor, or social cognition) based on a subjective and objective point of view and to a degree that does not interfere with every day instrumental activities and is not installed in the context of delirium or other mental disorder [2]. MCI can be further classified as amnestic or non-amnestic, keeping in mind the cognitive domains. Amnestic MCI refers to a pure impairment in recalling memorized information vs. non-amnesic MCI in which memory remains intact with the impairment of one or more other cognitive domains, other than learning and memory [3]. MCI is situated between unimpaired cognition and dementia, surpassing the natural decline by age in cognition but not meeting the criteria for dementia. The evolution of MCI is not always to dementia; in some cases, it reverses to the normal range of cognition correlated with age and gender [4].

The rapid growth in the global population and the prolonging of life expectancy contribute to an increasing number of elders, with those above 65 years growing in numbers faster than the younger population below that age. It has been estimated that by 2050 we will see a growth in the population of elderly people, from 10% in 2022 to 16% [5]. The prevalence of MCI in the elderly population is globally over 15%, varying from 8.7% in amnestic MCI to 10% in non-amnestic MCI, with a direct proportional rise with age and opposite to the level of education [6]. The progression of MCI to a type of dementia is estimated to be three to five times higher than those with normal cognitive function. Several risk factors have been identified, such as age, genetic heritage, education level, and gender [7].

There is no efficient drug therapy that decreases the risk of conversion of MCI to dementia. Attempts have been made with cholinergic inhibitors (rivastigmine, donepezil, galantamine), antihypertensive medication, steroids and over-the-counter supplements like vitamin B, C, D, E, calcium supplements, folates, with low effect or without sufficient data to conclude if there is any benefit. Regular physical exercise can have a mild beneficial effect on people with MCI [8,9]. MCI and its progression to dementia can produce chronic burdens on individuals and their families. In a 3-year longitudinal study, between 21.1 and 29.5% of caregivers have reported significant levels of burden, directly proportionally correlated with neuropsychiatric symptoms and lower functional ability [10].

Given the context (the evolution of MCI and the unsatisfactory therapies), it is essential to develop interventions that can delay or even prevent the onset of dementia and reverse MCI to normal cognitive function. Non-invasive brain stimulation has shown promising results, boosting synaptic plasticity and synaptic reorganization in healthy and affected patients [11].

### 1.2. Transcranial Direct Current Stimulation

Transcranial direct current stimulation is a transcranial electrical stimulation (tES) technique that uses a weak direct electrical current (1–2 mA) applied to the scalp via two or more electrodes, with the aim of modifying the brain function (neuromodulation). Anodal stimulation with tDCS (1–2 mA) by itself is not strong enough to depolarize the membrane potential of neurons to the firing threshold, and only increases the rate of spontaneous combustion and their excitability [12]. Conversely, cathodal stimulation is thought to deepen the resting membrane potential, making it difficult for neurons to depolarize, which reduces spontaneous combustion rates and the excitability of neurons. The increased excitability of local neurons by anodal stimulation is thought to increase blood flow around the stimulation site and induce subsequent metabolic changes. Specifically, blood flow changes through tDCS on the prefrontal cortex have been measured by functional near-infrared spectroscopy (fNIRS) [13].

Although many tDCS devices are capable of a range of stimulation intensities (e.g., 0–4 mA), most tDCS studies have used intensities of 2 mA or less and have elicited various measurable improvements [14]. However, if and how higher intensities might expand these outcomes have not been explored [15]. Recent studies have shown that intensities up to 4 mA are safe, tolerable, and do not elicit any serious adverse effects [14,16]. Now that the safety and tolerability of tDCS at higher intensities is better established, work exploring the performance differences between moderate (i.e., 2 mA) and higher (i.e., 4 mA) intensities is necessary to determine if increasing intensity further enhances outcomes [16]. Usually, the electrodes have a rectangular shape (5 × 5/5 × 7 cm) that are soaked in saline solutions or gels to increase the conductivity of the skin [17]. Placement of the electrodes on the head follows several standards such as: the international 10–20 (or 10-10) placement system, physiology-based system, neuronavigational system, and gross anatomical coordinates.

The location of the electrodes is a critical aspect of this treatment method, as it determines which brain regions are targeted and what effects are produced. tDCS typically involves the placement of two electrodes on the scalp—an anode (positive electrode) and a cathode (negative electrode). The anode is where the current enters the scalp, and the cathode is where it exits. Researchers carefully select electrode positions based on the desired cognitive or therapeutic effects. For example, the anode may be placed over a region that is associated with cognitive ability, while the cathode is placed elsewhere to complete this electrical circuit [17]. The selection of electrode placement in tDCS for MCI often targets brain regions associated with cognitive functions such as memory, attention, and executive function. Common areas of interest include the prefrontal cortex, which is involved in executive functions, and the dorsolateral prefrontal cortex (DLPFC), which plays a role in working memory and attention [18].

The optimal number of sessions of transcranial direct current stimulation (tDCS) for mild cognitive impairment (MCI) is an area of ongoing research and can vary based on several factors, including the specific tDCS protocol, the severity of MCI, and individual responsiveness. Different studies on tDCS for MCI have used varying numbers of sessions. Some studies have used as few as one or two sessions, while others have employed multiple sessions, ranging from five to twenty sessions or more [19].

The duration of a session varies, usually between 5 and 30 min; the longevity of the after-effects is proportional to the duration of the session. No additional benefits are seen beyond a 30-min-long session [20]. The lack of focality raises a major concern regarding tDCS, thus leading to the introduction of HD-tDCS [21]. High-definition transcranial direct current stimulation (HD-tDCS) uses smaller electrodes with a 4 mm radius (<5 cm^2^ total contact area) arranged in a circle with the purpose of increasing focality and restricting the stimulation mainly to the surrounding cortex. Five electrodes are typically used, the center and the surrounding electrodes having opposite polarities [17,21,22].

The mechanism of action is the modulation of membrane potential dependent on neuronal state. These techniques can only modulate the neuronal activity and do not induce action potentials in resting neurons; thus, the effect is dependent on the state of the targeted neuronal network [23]. Acute effects of direct current stimulation deriving from the modulation of neuronal resting potential are dependent on the orientation of the neuron relative to the orientation of electrical flow. Anodal stimulation increases depolarization while cathodal stimulation hyperpolarizes the resting potential, decreasing neuronal firing [24].

The belief that the effects of transcranial direct current stimulation (tDCS) may be attributed to placebo effects, or the power of suggestion is a common concern in the field of neuromodulation research. Some tDCS effects, such as changes in mood, pain perception, or cognitive performance, are inherently subjective and may be influenced by participants’ expectations and beliefs [25]. When individuals are aware that they are receiving a novel treatment, their perception of improvement can be influenced by the placebo response. In tDCS research, sham or placebo stimulation is often used as a control condition. Sham tDCS involves simulating the initial sensations of tDCS without delivering an actual current to the brain. Participants are often unable to distinguish between sham and active tDCS, and this can lead to the belief that the observed effects are solely due to placebo [26].

Long-standing after-effects are derived from changes in neuroplasticity. The glutamatergic synapse seems to be the principal actor influenced through the stimulation-induced increase in N-methyl-D-aspartate (NMDA) activity [27]. Another mechanism relies on the changes observed in GABA where anodal and cathodal stimulations decrease its concentrations [28,29]. These modifications lead to an increase in brain network connectivity resulting in an improvement of neuro-cognitive functions beyond the targeted cortical area [30,31]. While tDCS can have immediate and short-term effects, research on long-lasting effects is ongoing, and the extent of long-term benefits or changes is still a subject of investigation. While tDCS has shown promise in various applications, including cognitive enhancement, motor recovery, and mood regulation, there is limited research demonstrating long-lasting effects that persist beyond the immediate post-stimulation period [32]. Most studies have focused on the acute effects of tDCS. The duration and intensity of tDCS, as well as the number of sessions, can influence the potential for long-lasting effects. Prolonged or repeated stimulation may be more likely to lead to sustained changes in neural plasticity [33]. The extent of long-lasting effects can vary from person to person. Individual factors, including baseline brain function, responsiveness to tDCS, and the specific condition being treated, all play a role in determining whether and to what extent the effects persist [34].

Sex differences in transcranial direct current stimulation (tDCS) refer to the observed variations in the effects and responses to tDCS between males and females. Understanding these differences is essential for optimizing tDCS applications and tailoring treatments to individuals [35]. Sex differences can be influenced by biological factors, including hormonal fluctuations. For example, estrogen and progesterone levels in females can fluctuate during the menstrual cycle, which may impact brain excitability and, in turn, the effects of tDCS. Research has shown that males and females may respond differently to tDCS in terms of cognitive enhancement, pain perception, mood modulation, and other outcomes. Some studies have reported variations in the degree of response or even opposite effects for males and females [36]. There are anatomical differences in brain structure between males and females. These differences can influence the flow of electrical current through the brain, potentially leading to variations in the effects of tDCS based on electrode placement [37].

The integration of tDCS with modern neuroimaging techniques is likely to represent a key methodological approach for advancing our understanding of the functional correlates of tDCS mechanisms in terms of changes in patterns of brain activation and for understanding individual differences in response to stimulation [38,39]. Magnetic resonance imaging (MRI) is capable of providing high spatial resolution in terms of identifying anatomical regions. Functional MRI signal is often assumed to approximate the degree of local neural activation integrated over a spatial extent of several millimeters; however, this concept can be an oversimplification of the neurovascular coupling underlying many imaging sequences and more generally, the complex spatiotemporal properties of information processing [40]. Integration of tDCS with MR imaging techniques provides a tool to directly perturb neuronal function while monitoring brain state. Therefore, it enables researchers to study not only how stimulation modulates targeted brain regions, but also how tDCS modulates activity across the brain in the context of anatomical and functional connectivity [41]. In addition, this integration may also provide critical insights for understanding how, where and when stimulation is likely to be most effective—useful for optimization purposes.

Here, we review the studies investigating the effects of transcranial direct current stimulation (tDCS) and high-definition transcranial direct current stimulation (HD-tDCS) in patients with MCI.

## 2. Materials and Methods

The preferred reporting items for systematic review and meta-analysis (PRISMA) statement guidelines were used to conduct the literature search [42].

### 2.1. Search Strategy

Relevant studies were identified up to 25 May 2023 using four databases: PubMed, Web of Science, Scopus, and Embase. The search strategies used for the interrogation of databases was as follows: PubMed: ((transcranial direct current stimulation) OR (High definition transcranial direct current stimulation) OR (tDCS) OR (HD-tDCS)) NOT ((tACS) OR (tRNS) OR (magnetic) OR (deep)) AND ((mild cognitive impairment) OR (mild neurocognitive disorder)) AND ((clinical trial) OR (randomized controlled trial)) NOT (review) AND (2018:2023[pdat]); Web of Science: ((ALL = (transcranial direct current stimulation) OR ALL = (High definition transcranial direct current stimulation) OR ALL = (tDCS) OR ALL = (HD-tDCS)) NOT (ALL = (tACS) OR ALL = (tRNS) OR ALL = (magnetic) OR ALL = (deep)) AND (ALL = (mild cognitive impairment) OR ALL = (mild neurocognitive disorder)) AND (ALL = (clinical trial) OR ALL = (randomized controlled trial)) NOT ALL = (review)); Scopus: (transcranial AND direct AND current AND (&apos;stimulation&apos;/exp OR stimulation) OR (high AND (&apos;definition&apos;/exp OR definition) AND transcranial AND direct AND current AND (&apos;stimulation&apos;/exp OR stimulation)) OR tdcs OR &apos;hd AND tdcs&apos;) AND NOT (tacs OR trns OR magnetic OR deep) AND (mild AND cognitive AND (&apos;impairment&apos;/exp OR impairment) OR (mild AND neurocognitive AND (&apos;disorder&apos;/exp OR disorder))) AND ((&apos;clinical&apos;/exp OR clinical) AND (&apos;trial&apos;/exp OR trial) OR (randomized AND controlled AND (&apos;trial&apos;/exp OR trial))) AND NOT (&apos;review&apos;/exp OR review) AND (LIMIT-TO (PUBYEAR, 2023) OR LIMIT-TO (PUBYEAR, 2022) OR LIMIT-TO (PUBYEAR, 2021) OR LIMIT-TO (PUBYEAR, 2020) OR LIMIT-TO (PUBYEAR, 2019) OR LIMIT-TO (PUBYEAR, 2018)); Embase: (transcranial AND direct AND current AND (‘stimulation’/exp OR stimulation) OR (high AND (‘definition’/exp OR definition) AND transcranial AND direct AND current AND (‘stimulation’/exp OR stimulation)) OR tdcs OR ‘hd tdcs’) NOT (tacs OR trns OR magnetic OR deep) AND (mild AND cognitive AND (‘impairment’/exp OR impairment) OR (mild AND neurocognitive AND (‘disorder’/exp OR disorder))) AND ((‘clinical’/exp OR clinical) AND (‘trial’/exp OR trial) OR (randomized AND controlled AND (‘trial’/exp OR trial))) NOT (‘review’/exp OR review) AND [2018–2023]/py.

### 2.2. Inclusion and Exclusion Criteria

#### 2.2.1. Population

Clinical studies involving adults with MCI were included. MCI was defined either by the criteria of Petersen et al.: (1) abnormal function of the memory, (2) more than in normal ageing, (3) that does not impair general cognitive function and daily living activities, (4) the patient being self-aware and complaining about the deficit, in (5) the absence of dementia [1], or by DSM-V criteria: (1) a “Mild Neurocognitive Disorder” with the specification that the cognitive decline from a previous level of function in 1 or more of 6 cognitive domains (complex attention, executive function, learning and memory, language, perceptual motor, or social cognition) is based on a subjective and objective point of view and does not interfere with every day instrumental activities and is not installed in the context of delirium or other mental disorder [2].

#### 2.2.2. Intervention

tDCS was defined as a technique that uses a weak sustained direct electrical current (1–2 mA) applied to the scalp via two or more electrodes, with the aim of modifying the brain function in the cognitive domain.

#### 2.2.3. Study Design

Only primary research studies were included, represented by randomized controlled trials, and non-randomized controlled trials. Reviews, case-reports, editorial reports, and conference abstracts were excluded.

#### 2.2.4. Outcome

Studies had to report variations in one or more cognitive domains to be included in this study.

Secondary outcomes such as sleep, pain, or affective domains could be also included.

### 2.3. Selection Process

The studies identified by the search query adapted for each database were first screened using the title and the abstract. Studies were included or excluded based on the criteria defined above. No language restriction was used. The included studies by abstract were rescreened by reading the full text. Two reviewers, M.P. and O.D.C., independently assessed the interrogation results. Disagreements regarding the inclusion/exclusion of an article were resolved with discussion and debate with the whole team.

Relevant data were extracted and synthesized in a standardized data table included in this review.

## 3. Results

**Figure 1:**
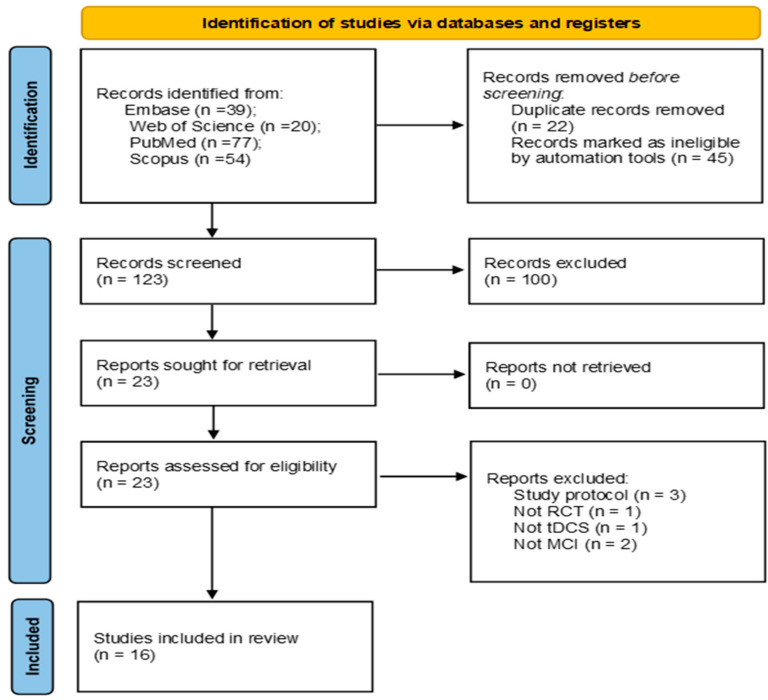
PRISMA flowchart.

Figure 1 summarizes the selection process of studies screened at each stage. A total of 190 studies were identified from database interrogation. From these, 67 were excluded by automation tools being duplicates or non-RCT (conference abstracts, letter to the editor, and editorials). Furthermore, out of 123 records automatically screened, 100 were excluded based on the title and the abstract, resulting in 23 eligible papers. Seven more papers were rejected after a full-text lecture with the reasons listed in the figure below. Finally, 16 studies were included in this review.

### 3.1. Transcranial Direct Current Stimulation on Cognition

Fileccia et al. (2019) [43] evaluated the effects of tDCS with the active electrode over the left dorsolateral prefrontal cortex (LDLPFC) on patients with MCI compared to sham. The study design was a single-blinded parallel RCT with two groups (17 active vs. 17 sham) of MCI patients. The patients were neuropsychologically assessed before and after the intervention. The battery of tests was composed of the Mini Mental State (MMSE); Brief Mental Deterioration Battery (BMDB); Rey Auditory Verbal Learning Test (RAVLT): immediate recall and delayed recall; Immediate Visual Memory; Copy Design: simple; Barrage test; Stroop test; verbal fluency: phonemic and semantic; naming to description; figure naming, analogies; State Trait Anxiety Inventory-Y and the Beck Depression Inventory. The stimulation protocol consisted of 20 min 2 mA anodal active stimulation, 5 days per week, and 20 days of stimulation in total. The two groups did not differ at baseline assessment in age, sex, or education. In the results after 20 days of stimulation, the patients in the active group compared to baseline and sham stimulation had a significantly better performance in BMDB, RAVLT: immediate recall, figure-naming test, and the Beck Depression Inventory. A trend of improvement was seen in MMSE, Immediate Visual Memory, the Barrage test, and Rey’s delayed recall [43]. One limitation of this study is the lack of multiple measurements in time, with no follow-up on long-term after-effects.

Stonsaovapak et al. (2020) [44] applied multiple sessions of anodal tDCS over the right DLPFC on individuals with MCI. The study design was a parallel double-blind RCT; the control group received sham stimulation. The total number of sessions was 12, three times a week for 4 weeks, each session duration was 20 min. The intensity of the direct current was 1 mA. The baseline cognitive status was assessed before stimulation. Three more assessments after stimulation were effectuated: one immediately after the first stimulation, one after 4 weeks, and one post-session at week 8. The cognitive changes before and after intervention were evaluated using the Cambridge Neuropsychological Test Automated Battery (CANTAB) assessing rapid visual information processing (RVP), visual sustained attention (VSA), spatial working memory (SWM), delayed matching to sample (DMS) and pattern recognition memory (PRM). In the active group, the results showed a significant increase in the RVP and DMS scores and a significant decrease in SWM, DSM, and PRM scores at all three measured time points. Comparing the sham and active groups, tDCS showed a significant effect on RVP total hits, SWM total errors, and DMS scores. RVP total hits improvement was seen at all three time points, while SWM between errors/total errors and DMS total corrected scores decreased immediately after the first stimulation but were not maintained at weeks 4 and 8. One limitation of this study was the inability to eliminate the learning effect of the automated battery of tests [44].

Manenti et al. (2020) [45] performed a double-blinded parallel RCT on patients with amnestic mild cognitive impairment (aMCI). Active anodal tDCS was applied over the left lateral prefrontal cortex (PFC) for 15 min using 1.5 mA for one session. The experimental protocol had multiple steps: step 1, learning of 20 words; after 24 h on step 2, the patients were actively or sham-stimulated after a contextual spatial reminder; on step 3, on day 3 and day 30, the participants were asked to recall the learned words in step 1. If the participant stopped free recalling words, the experiment switched to a recognition task. The results showed that active tDCS does not enhance free word recall but does enhance word recognition compared to the sham group. The effect diminished at day 30, with no differences between groups. The limitations of this study are the relatively small sample size, no pre-stimulation memory performance was assessed, and some intergroup variability may be present [45].

Gu et al. (2022) [46] examined the effect of tDCS over episodic memory on individuals with MCI in a double-blinded parallel RCT. The patients were divided into two groups, sham and active tDCS. The active anodal electrode was placed over the left temporal area. The stimulation duration was 20 min per day for 5 days consecutively, using 2 mA. They assessed the outcomes using the Montreal Cognitive Assessment Scale (MoCA), Wechsler memory scale (WMS), and event-related potential (ERP) (amplitude and latency of P300 wave). The assessment was performed at three time points: pre-treatment, 5 days, and 4 weeks post-treatment. The result revealed that a significant improvement in Memory Quotient score (MQ), picture memory, visual regeneration, logical memory, memory span, and visual regeneration/memory delay in the active tDCS group after 5 days of stimulation. This improvement was maintained at the 4 week post-treatment revaluation, with no significant score difference between day 5 post-intervention. The ERP P300 wave had a significant increase in amplitude with a shorter latency after 5 days and 4 weeks post-intervention. This proves that tDCS can improve episodic memory. The limitation of this study was the small sample size and short follow-up period of time [46].

Aksu et al. (2022) [47] conducted a study to investigate the effect of tDCS on patients with Parkinson’s disease MCI. The active anodal electrode was placed over left DLPC, for 20 min with an intensity of 2 mA. Ten sessions were administered, twice a day for 5 days. The outcome was assessed using a battery of neuropsychological and electrophysiological evaluations, immediately and 1 month after intervention. The results showed a significant improvement in the delayed recall domain and executive function immediately after intervention; even more, the improvement was maintained after 1 month. No statistically significant electrophysiological changes were observed between the groups. One principal limitation of this study is the small sample size and the short duration of follow-up [47].

Table 1 summarizes these findings.

### 3.2. Transcranial Direct Current Stimulation Combined with Cognitive Training

Lawrence et al. (2018) [48] applied tDCS alone or combined with standard cognitive training (SCT) and tailored cognitive training (TCT) to patients with Parkinson’s disease (PD) and MCI compared to controls with no intervention, standard cognitive training, or tailored cognitive training alone. Cognitive training was delivered by Smartbrain Pro which trained each cognitive domain. In the tDCS + SCT and SCT groups, a predetermined program was used. In tDC + TCT and TCT, the training activities were individualized depending on the neuropsychological baseline. The tDCS was applied for 20 sessions and each session duration was 20 min with an intensity of 1.5 mA. The placement of the anodal electrode was over the dorsal lateral prefrontal cortex. The neuropsychological domains evaluated were: executive function, attention and working memory, memory, visuospatial abilities, and language. The results showed in the SCT group a memory improvement, activities of daily living, and quality of life; in tDCS alone an improvement in attention, working memory, and memory; in SCT + tDCS showed an improvement in executive function, attention, working memory and activities of daily living; in the TCT + tDCS, an improvement on executive function, attention, working memory, and memory [48]. The following short period of time can be a limitation of this study.

Das et al. (2019) [49] examined the effects of tDCS combined with Gist reasoning training (SMART) on individuals with MCI. The patients were randomly assigned to tDCS + SMART or Sham + SMART. tDCS stimulation was delivered over the left inferior frontal gyrus for 20 min with an intensity of 2 mA, before SMART. The SMART session was coupled either with tDCS or Sham-tDCS for a total of 8 sessions during 4 weeks. They also assessed the effects of the intervention over resting cerebral blood flow with an MRI scan. The results showed a significant improvement in the active stimulation group over the executive function of inhibition and innovation and episodic memory measured after immediate intervention. However, the cognitive improvements did not last until the follow-up measurements at 3 months. An increase in resting cerebral blood flow was observed at the right middle frontal cortex in the active group, with no relationship with neurocognitive findings [49].

Martin et al. (2019) [50] investigated the effect of tDCS combined with cognitive training (CT) on patients with amnestic mild cognitive impairment. They applied 15 sessions of tDCS + CT or tDCS + Sham. Active anodal tDCS stimulation was placed over the left dorsolateral prefrontal cortex for 30 min with 2 mA. CT was administered using COGPACK training memory and learning abilities for approximately 45–60 min. The primary outcome measurements comprised the Total Learning T score on the California Verbal Learning Task, second edition, a non-trained test of verbal memory. The results showed a significant improvement from baseline in both groups for any outcome. After 3 months follow-up, significant improvements in verbal memory, visual memory, attention and working memory, processing speed, and subjective cognitive functioning were shown compared to the pre-treatment status in both groups. Also, an improvement in mood and instrumental activities of daily living was observed in both groups on follow-up [50]. Some limitations of this study are the sample size and, the combination of two techniques which can affect the treatment outcome.

Lu et al. (2019) [51] compared the effects of tDCS combined either with working memory training (WMT) or using cognitive training and Sham-tDCS + WMT. They applied 12 sessions during 4 weeks, of 45 min each. The anodal active tDCS was delivered over the left lateral temporal cortex for 20 min with 2 mA intensity. The primary outcome measurement was the working memory test assessed by N-back test and global cognitive function assessed by the Alzheimer’s Disease Assessment Scale-Cognitive Subscale (ADAS-Cog). The secondary outcome’s measurements assessed memory and language, neuropsychiatric events and potential adverse effects of the therapy. Working memory training was assessed using the Adaptative N-back test using E-prime 2.0. A continuous performance test was used for cognitive training, performed using the same tool as in WMT. The outcome measurements showed a significant improvement in global cognition measures with no difference between groups, with a trend to decrease to baseline after 12 weeks. N-back performance task improvements were shown in all groups, persisting after 12 weeks post-intervention. The secondary outcomes showed an improvement in memory and language immediately after the intervention, with only tDCS + WMT retaining the effects on delayed recall after 12 weeks [51]. A limitation of this study is the lack of controls for each intervention.

Gonzalez et al. (2021) [52] investigated the effect of tDCS coupled with CT compared to sham-DCS + CT and CT alone. All participants performed nine sessions of training in 3 weeks, three sessions per week. tDCS stimulation was performed for 30 min using an intensity of 1.5 mA; the active anodal electrode was placed over the left dorsolateral prefrontal cortex. Cognitive training was performed using the Neuron Up online platform; the duration of one session was 30 min. The primary outcomes measured were cognitive domain-specific as follows: MoCa, Digit Span Test, Trail Making Test. The secondary outcome measured included the Rivermead Behavioral Memory Test. The results showed no superiority on primary measurements of tDCS + CT across the compared groups with no significant differences between groups after intervention and on follow-up. The combination of tDCS and CT improved processing speeds [52].

Rodella et al. (2022) [53] combined tDCS with computerized cognitive training for 12 sessions during 4 weeks; each session duration was 45 min. The electrical stimulation duration was 30 min using 2 mA and the anodal electrode was placed over the left dorsolateral prefrontal cortex. The cognitive training was delivered using CoRe software which targeted logical-executive, processing-speed, working memory, and episodic memory functions. The global cognitive function was the primary outcome measured. The secondary outcome assessed was other cognitive domains, via performance in the CoRe tasks. The results measured after the end of sessions showed a significant improvement in working memory, attention and processing speed in the active tDCS + CT group vs. sham-tDCS + CT group. After 6 months follow-up, the assessments showed the maintenance of the beneficial effect over working memory in the actively stimulated group with a trend to significance of attention/processing speed. More than that, the MMSE score did not decline in the active group compared to the sham group in which it worsened after 6 months [53]. These results show a positive trend, despite all the studies showing a nonmajor effect of tDCS combined with CT.

All the effects above are synthetically refined in Table 2.

### 3.3. Transcranial Direct Current Stimulation Combined with Dual-Task Gait Training

Lioa et al. (2021) [54] investigated the effects of tDCS combined with Tai Chi on cognitive function and dual-task gait performance. The patients followed a 3-week training period, 3 times a day for 36 sessions. Active anodal tDCS stimulation was delivered over the left dorsolateral prefrontal cortex with an intensity of 2 mA for 20 min. Tai Chi training was delivered by a professional trainer for 40 min. The primary outcome measured was gait performance measured by Gait UP system under three conditions: single task (normal walking); the cognitive dual-task (while performing a cognitive task); the motor dual-task (while carrying a tray with glasses). The secondary outcome measured was the cognitive task performance, visual working memory, planning and problem-solving capacity, visual attention, and verbal learning. The results showed a significant interaction regarding cadence and speed in cognitive dual-task performance with no cognitive task performance improvements in both active and sham groups [54].

### 3.4. Effects on Neuropsychiatric Symptoms

Turnbull et al. (2023) [55] evaluated the effects of tDCS on MCI patients over neuropsychiatric symptoms (NPS) during visual object tracking. The placement of the electrode was over the left sensorimotor cortex (LSMC). The intensity of the current was 2 mA applied for 20 min. The visual object tracking task duration was 20 min. The intervention has 20 sessions in total. They collected behavioral and neuroimaging data immediately after the intervention and at 4 weeks afterwards. The NPS were measured using two perspectives: a self-reporting scale for depression, anxiety, apathy; and a caregiver-reported scale using the Neuropsychiatric Inventory with 12 domains. The neuroimaging data were acquired using an MRI scanner. The results showed an improvement in self-reported mood immediately after intervention with no changes in caregiver reports. The MRI scan showed changes in LSMC activation, frontal cortex and the left amygdala, which can be correlated with mood changes [55].

### 3.5. Effects on Visuospatial Memory

De Sousa et al. (2020) [56] evaluated the effects of tDCS over object-location memory (OLM) on individuals with MCI in a crossover RCT. The associative memory training sessions were combined with tDCS. The patients had to remember the locations of 30 objects placed on a 2D map. The tDCS session duration was 20 min at 1 mA intensity with the anodal electrode placed over the right temporal cortex. The measurement was collected 1 day and 1 month after training completion. The results showed a small benefit in training success in active stimulated patients compared to sham immediately after training. On long-term memory, no substantial effect was observed. No sustained positive effect was found for delayed memory after 1 month [56].

### 3.6. Effects over the Theory of Mind

Adenzato et al. (2019) [57] investigated the effects of tDCS on patients with Parkinson’s disease (PD) and MCI compared to healthy controls over the theory of mind (ToM). They applied one session of tDCS over the medial frontal cortex (MFC) for 6 min and one sham session for each participant. Each session was separated by at least two days of pause. Two tasks were evaluated, Reading the Mind in the Eyes (RME) (the ability to describe the mental state of the other only by observing their eyes) and Attribution of Intention (AI) (choosing the right result from a dichotomic ending of a video story). The results showed a shortening in latency of RME and AI tasks with no effect on accuracy in PD-MCI patients, a direct proportional relation between executive function and ToM tasks (patients with frontal executive impairment performed poorer) and the healthy controls performed better than the impaired participants [57].

### 3.7. Effects on Spatial Navigation

Iordan et al. (2022) [58] assessed the effects of HD-tDCS on allocentric and egocentric navigation and brain network segregation. The electrical stimulation was focused over the right superior parietal cortex, with an intensity of 2 mA for 20 min. The navigation task was performed simultaneously with a fMRI scan after electrical stimulation. The results showed no significant effect on performance in allocentric and egocentric navigation tasks. In MCI patients, active stimulation, increased overall network segregation, andin sensory-motor, association system network, dorsal-attention and default mode networks segregation to a level similar to the healthy control group. These findings suggest that this technique can be useful to normalize the brain network segregations in patients with MCI [58].

Overall, the studies described in this paper suggest that active tDCS over the prefrontal cortex can improve the cognitive symptomatology in MCI. Huo et al., in a meta-analysis from 2019, found that tDCS can ameliorate the decline in memory in older individuals with an improvement immediately after stimulation and at long-term follow-up [59]. In contrast, the results of a more recent meta-analysis suggest that tDCS has no significant effect on cognition regarding global functioning, memory, sustained attention, and executive function in MCI patients [60].

The stimulation outcome may be affected by stimulation polarity (cathodal/anodal), the site on which the electrodes are placed, the number of sessions and other training effectuated during stimulation [61]. Stimulation polarity may play an important role in the global effect, in a recent meta-analysis by Saxena and Pal in 2021 on AD patients, have concluded that the effect of anodal tDCS is superior to cathodal stimulation [62]. However, considering that cathodal stimulation might have no effect may be wrong. In a rodent model of AD study that investigated the difference between the two types of stimulation, it was found that both anodal and cathodal stimulation improved spatial learning and/or memory, decreased delta band EEG activity in the prefrontal cortex, and increased it in gamma band. In particular, anodal stimulation increased alpha band prefrontal cortex activity spontaneously while cathodal stimulation increased alpha-beta activity in task performance state [63]. There might be a similar mechanism involved in the effects of anodal and cathodal stimulation, despite the expectation that opposite polarity of stimulation causes opposite effects. Future studies are required to elucidate this question.

The placement of the electrodes may have an important role; intuitively, we aim at the temporal lobes which are an important hub for learning, recall and recognition. It has been stipulated that the left temporal volume is a negative correlation risk factor of MCI progression to dementia [64]. The volume of the hippocampus is also a negative correlation risk factor of progression to AD, having an important role in short- and long-term memory [45,46]. Because of the placement of the hippocampus, deep in the brain, the direct influence of tDCS is impossible. Also, the prefrontal cortex is impacted in MCI affecting language, executive function, attention, and memory [65]. DLPF plays an important role in the processing of various functions such as working memory, attention, planning and problem-solving, thus being a target for most of the studies included in this paper.

In his network meta-analysis, Tseng et al. found that a cathode placed over F3 (DLPFC) and an anode over Fp2 (orbito-frontal) is associated with significant positive effects over cognition and cognitive decline in patients with dementia compared to sham [66]. In contrast, another recent meta-analysis found that stimulation of temporal lobe-related area improves the overall cognitive functions in patients with MCI better than stimulation of DLPFC [67]. This dichotomy of results reflects the fact that a multicentric study is needed with a vigorous methodology.

The optimal number of sessions and the time period between them remain to be established in future studies. In healthy people, experiments showed that the effect of a single session of stimulation can be observed after a day; meanwhile, after that period, the aftereffects are negligible [68].

All of the above-described effects are summarized in Table 3.

## 4. Discussion

The combination of tDCS with other training might bring supplementary beneficial effects. Pallanti et al., 2022 [69] combined the stimulation sessions with cognitive training observing significant improvement in global cognitive function and verbal fluency [69]. tDCS has the potential to slow down the progression of MCI to AD by improving cognitive functions, reducing amyloid plaques, improving cerebral blood flow, increasing neuronal plasticity, regulating neurotransmission, reducing excessive glial activation and inhibiting neuroinflammation [70]. This shows that in the future, well-designed randomized studies combined with other cognitive therapies will be expected to determine the optimal stimulation path.

It is important to acknowledge the limitations. In most of the studies, the sample size is too small, thus the statistical power is low and influences the conclusions. A short period of follow-up evaluation means data about long-term changes are not available. All the studies did not evaluate in vivo bio-markers which give us information about neurodegenerative status pre- or poststimulation. For example, it has been suggested that both plasma p-tau181 and p-tau231 are increased in cognitively unimpaired APOE4 carriers, being an early marker in AD [71]. Also, neuroimaging can be a useful tool to measure the brain status before and after stimulation, thus helping to understand the effects and to guide the therapy. Measuring biological changes induced by the intervention may explain the mechanism of behavioral changes and guide the therapy.

A relevant discussion can also refer to the difference between responders and non-responders. In this way, according to our best knowledge, there have been no relevant studies regarding this subject until the present; this might be the result of negative results study bias. This dichotomy can have a key role in understanding the mechanisms of successful vs. non-successful ageing; in the future, guiding therapy and improving the outcomes.

A difference in neuronal activation correlated with age was described by Cabeza et al. (1997), namely HAROLD (Hemispheric Asymmetry in Older adults). This accounts for the fact that elders compensate for their cognitive impairment with a greater bilateralism compared to younger adults. This pattern is associated with greater cognitive outcomes by compensation, and a decrease in network segregation [72]. Vecchio et al. researched the effects of tDCS on network properties, using SW (small word) as a model of organization, with normal aging people **vs.** young individuals, delivered over the prefrontal cortex. SW was defined as the balance between global integration and local specialization. It was observed that tDCS increased SW in alpha similar to a reduction in delta and theta frequency band in both groups actively stimulated compared to the sham group. An interesting difference between young and elderly individuals is that the polarity of stimulation did not affect the outcome in the last group; this can show a loss of a mechanism of brain reactivity [73]. Francesca et al. showed that anodal tDCS applied to the posterior parietal cortex has a greater effect on SW parameters compared to prefrontal cortex stimulation in younger adults, but neither of the types of stimulation had an effect on older adults. This can show an alteration in cognitive and plasticity mechanisms during normal ageing [74]. A more focal stimulation therapy has emerged, HD-tDCS, but has not yet been sufficiently researched. In our paper, we included one study that assessed the effects of HD-tDCS over allocentric and egocentric navigation and brain network segregation with no significant effects in these domains. An increase in sensory-motor, association system network, dorsal-attention and default mode networks segregation has been observed to a level similar to the healthy control group, suggesting that this technique can be useful to normalize the brain networks segregations in patients with MCI [58]. The HAROLD hypothesis may explain these findings, with the effects of tDCS on brain organization being age-related.

Via its characteristics, MCI opens the path of so-called disease-modifying therapies, such as anti-amyloid monoclonal antibodies [75]. A combination of these substances with tDCS may be a good approach to slow the degradative progression or even more to reverse it. Nevertheless, the lack of publication of negative results influences scientific progress, leaving the path of redundancy open.

## 5. Conclusions

The growing body of work suggests that tDCS may be a useful tool in neuromodulation of cognitive function and may promote a successful cognitive ageing [76]. This result awaits replication and translation in clinical practice. More studies are expected for a better understanding of the underlying mechanisms, therapy outcomes, the variables that influence it and to optimize the stimulating parameters. Future studies are needed to investigate and establish the optimal stimulating parameters such as dose, time of a session, time between sessions, optimal stimulating site and the optimal combination with other drugs and techniques that can change the progression of the disorder. Despite its positive feedback from clinical trials, we must understand that tDCS is still an unevolved subject, and much more research needs to be completed in order to discover its long-term safety. Furthermore, medical professionals must receive proper training in the technique and adhere to all the safety guidelines when using tDCS.

## Figures and Tables

**Table 1:** tDCS in patients with mild cognitive impairment (MCI).

Study ID	Design	Control Condition	Sample		tDCS Characteristics			tDCS Outcome
			Participants	Age tDCS vs. Control (Mean ± SD)	Electrodes (Surface/Location 10–20 International System)	Sessions (Minutes/Sessions)	Intensity mA	
Fileccia et al., 2019 [43]	single-blinded parallel RCT	Sham	*n* = 34 17 vs. 17	71.6 ± 1.4 vs. 69.7 ± 1.6	Anode: 7 × 5 cm Cathode: 7 × 6 cm/ Anode: F3 (left DLPFC) Cathode: right deltoid muscle	20/20	2	Improvement in Brief Mental Deterioration Battery, figure naming test and Beck Depression Inventory. A trend to improvement in MMSE, immediate visual memory, barrage test and Rey’s 15-word delayed recall.
Stonsaovapak et al., 2020 [44]	double-blinded parallel RCT	Sham	*n* = 55 23 vs. 22	68.39 ± 8.37 vs. 69.68 ± 7.60	5 × 5 cm/ Anodal: F4 (right DLPFC) Cathode: left supraorbital area	20/12	2	Immediate effect: significant increase in RVP total hits and DSM total score, decrease in SWM between errors and SWM total errors (effect not maintained after 4 and 8 weeks post-therapy). After 8 weeks, a significant decrease in the DSM and PRM latency post-therapy was observed.
Manenti et al., 2020 [45]	double-blinded parallel RCT	Sham	*n* = 18 9 vs. 9	75.3 ± 4.8 vs. 75.3 ± 2.2	7 × 5 cm/ Anodal: F3(left LPFC) Cathode: FP2(right supraorbital)	15/1	1.5	May enhance word recognition but not free recalling. Decrease in the effect on day 30 after stimulation.
Gu et al., 2022 [46]	double-blinded parallel RCT	Sham	*n* = 40 20 vs. 20	63.20 ± 6.98 vs. 65.15 ± 6.16	7 × 5 cm/ Anode: T3(left temporal area) Cathode: right shoulder deltoid muscle	20/5	2	After 5 days: improvement in MQ score, picture memory, visual regeneration, logical memory, memory spam, visual regeneration-delay and logical memory-delay. 4 weeks later, the effect was maintained. Increase in amplitude and decrease in latency of P300 wave after 5 days of therapy with maintenance after 4 weeks.
Aksu et al., 2022 [47]	double-blinded parallel RCT	Sham	*n* = 26 13 vs. 13	65.52 ± 7.49	5 × 7 cm/ Anodal: F3(left DLPFC) Cathodal: F4(right DLPFC)	20/10	2	Significant immediate improvement in executive function and delayed recall domain with an important improvement in overall cognitive function after 1 month.

DLPFC—dorsolateral prefrontal cortex; DMS—delayed matching to sample; LPFC—lateral prefrontal cortex; MMSE—mini-Mental State Examination; MQ—memory quotient; PRM—pattern recognition memory; RVP-rapid visual information processing; SWM-spatial working memory.

**Table 2:** tDCS combined with cognitive training.

Study ID	Design	Control Condition	Sample		tDCS Characteristics			tDCS Outcome
			Participants	Age tDCS vs. Control (Mean ± SD)	Electrodes (Surface/Location 10–20 International System)	Sessions (Minutes/Sessions)	Intensity mA	
Lawrence et al., 2018 [48]	parallel RCT	No intervention	*n* = 42 5 × 7 vs. 7	68.14 ± 8.69 vs. 65.57 ± 5.20 vs. 72 ± 6.45 vs. 63.57 ± 15.68 vs. 67.43 ± 6.37 vs. 72.29 ± 6.21	5 × 7 cm/ Anode: F3 (left DLPFC) Cathode: above the left eye	20/4	1.5	tDCS group improved on working and attention memory, and memory. CT + tDCS group improved on executive function, attention, working memory and ADL. Tailored CT + tDCS group improved on executive function, attention, working memory and memory.
Das et al., 2019 [49]	double-blinded parallel RCT	Sham + SMART	*n* = 22 12 vs. 10	62.58 ± 8.43 vs. 63.30 ± 7.38	3 × 5 cm/ Anode: 0.5 cm above left eyebrow and 1 cm away from the center of the nasal bridge Cathode: right shoulder	20/8	2	Immediate cognitive improvement in sham group executive function of inhibition, innovation and episodic memory. Significant increase in cerebral blood flow in the middle frontal cortex in the active group.
Martin et al., 2019 [50]	double-blinded parallel RCT	CT + Sham	*n* = 68 33 vs. 35	71.8 ± 6.39 vs. 71.6 ± 6.35	5 × 7 cm/ Anode:F3(left DLPC) Cathode: F8)	30/15	2	No significant difference between CT + sham and CT + tDCS
Lu et al., 2019 [51]	double-blinded parallel RCT	tDCS + WMT/CT vs. Sham-tDCS + WMT	*n* = 201 69 vs. 68 vs. 64	74.2 ± 6.7 vs. 73.4 ± 6.1 vs. 74.5 ± 6.6	5 × 7 cm/ Anode: T3 (left LTC) Cathode: right upper limb	20/12	2	Improvement due to WM and CT training with no defervescences between groups on ADAS-Cog score, N-BACK test and RT. All groups showed improvement in delayed recall and working memory with the greatest benefit on tDCS-WMT group.
Gonzalez et al., 2021 [52]	double-blinded parallel RCT	sham tDCS + CT group/CT group alone	*n* = 67 22 vs. 24 vs. 21	69.8 ± 5.3 vs. 71.0 ± 6.2 vs. 70.6 ± 5.4	5 × 3 cm/ Anodal:F3(left DLPFC) Cathode: right brachioradialis muscle	30/9	1.5	The association of CT + tDCS can improve the processing speed related to working memory and attention. No superior effects compared to sham + CT or CT alone.
Rodella et al., 2022 [53]	double-blinded parallel RCT	Sham + CoRE	*n* = 33 16 vs. 17	71.62 ± 5.65 vs. 75.13 ± 4.76	Anode: 16 cm^2^ Cathode: 50 cm^2^ / Anode: F3 (DLPFC)Cathode: right deltoid	30/12	2	After intervention: significant improvement in working memory, attention, processing speed; no significant difference in MMSE, episodic long-term memory, logical-executive function. No improvement in control group. After 6 months: significant improvement in working memory and an ascension toward significance in attention and processing speed; no significant difference in MMSE, episodic long-term memory, logical-executive function. Long-lasting improvement in working memory, attention and processing speed in the active arm.

ADL—activities of daily living; CT—cognitive training; CoRE—computerized cognitive training; DLPFC—dorsolateral prefrontal cortex, LTC-lateral temporal cortex, MMSE—mini-Mental State Examination; RT—reaction time; SMART—Gist reasoning training; WMT—working memory training.

**Table 3:** Effects of tDCS in various fields.

Study ID	Design	Control Condition	Sample		tDCS Characteristics			tDCS Outcome
			Participants	Age tDCS vs. Control (Mean ± SD)	Electrodes (Surface/Location 10–20 International System)	Sessions (Minutes/Sessions)	Intensity mA	
Lioa et al., 2021 [54]	double-blinded parallel RCT	Sham + Tai Chi	*n* = 20 10 vs. 10	72.6 ± 4.1 vs. 73.1 ± 4.6	5 × 7 cm/ Anodal: F3 (left DLPFC) Cathodal: F4 (right supraorbital area)	20/36	2	Significant increase in speed and cadence and increase in speed of cognitive dual-task gait. No significant cognitive task performance improvement.
Turnbull et al., 2023 [55]	double-blinded parallel RCT	Sham + MOT	*n* = 40 20 vs. 20	70 ± 6.9 vs. 73 ± 7.1	30 cm/ Anodal: C3 (LSMC) Cathodal: Fp2 (orbitofrontal)	20/7	1.5	Immediately self-reported mood improvement from baseline/no improvement reported by caregivers. Decrees in LSMC activation; increase activation on post-central gyrus, LSMC-amygdala FC.
de Sousa et al., 2020 [56]	single-blinded crossover RCT	Healthy patients	*n* = 48 16 vs. 32	70 ± 6 vs. 69 ± 7	Anode: 5 × 7 cmCathode: 10 × 10 cm/ Anode: T6 (right temporoparietal cortex) Cathode: supraorbital area	20/3	1	Small benefit on short and long-memory recall; small benefit for patients with MCI; no benefit for HP in training success. No substantial effect on long-term memory. Improvement in OLM training + tDCS in HP and MCI patients. Small impact over sleep characteristics. MCI patients scored somewhat higher on affective state regarding “excitation and anxiety”.
Adenzato et al., 2019 [57]	double-blinded crossover RCT	Sham/HC	*n* = 40 20 vs. 20	65.6 ± 8.4 vs. 69.4 ± 5.8	7 × 5 cm/ Anode: Fpz (MFC) Cathode: between Inion and OZ	6/1	1.5	Stimulated MCI patients have a decrease in latency in theory of mind tasks.
Iordan et al., 2022 [58]	double-blinded crossover RCT	Sham	*n* = 42 20 vs. 22	72.15 ± 7.14 vs. 69.50 ± 6.55	No surface mentioned/ Anode:p2 Cathode: CPz, CP6, POz, PO8	2/20	2	No significant behavioral effect on performance in allocentric or egocentric navigation. May normalize whole brain network segregation during task performance. Increased segregation between the associating networks inducing a response in sensory–motor system; increase segregation in dorsal-attention and default-mode networks.

DLPFC—dorsolateral prefrontal cortex; FC—functional connectivity; HP—healthy patients; LSMC—left sensorimotor cortex; MFC—medial frontal cortex; OLM—object-location memory.
